# HER 2/neu protein expression in colorectal cancer

**DOI:** 10.1186/1471-2407-6-123

**Published:** 2006-05-08

**Authors:** B Schuell, T Gruenberger, W Scheithauer, Ch Zielinski, F Wrba

**Affiliations:** 1 Department of Internal Medicine I, Division of Clinical Oncology, University Hospital, Vienna, Austria; 2 Department of Surgery, University Hospital, Vienna, Austria; 3 Department of Pathology, University Hospital, Vienna, Austria

## Abstract

**Background:**

Conflicting data exist about the prevalence of HER-2/neu overexpression in colorectal cancer ranging from 0 to 83 %. In our study we tried to clarify the extent of expression and its relationship to clinicopathological parameters.

**Methods:**

This study involved 77 specimens of malignant colorectal cancer lesions of surgically resected patients. HER-2/neu immunohistochemistry was performed using the Hercep-Test Kit.

**Results:**

Out of 77 specimens, 56 were Her-2/neu negative (70%), 20 (26%) showed a barely immunostaining (1+), only 1 (1%) was moderately (2+) and 2 (3%) were strongly positive (3+). Her-2/neu staining (moderately and strongly positive) was only detected in primary tumours of patients with confirmed metastases. No relationship was found between membranous HER-2 expression and patients' gender or differentiation. The median survival time of patients with positive HER-2/neu immunostaining was 21 versus 39 months in patients without HER-2/neu expression (p = 0.088).

**Conclusion:**

The c-erbB protein expression was observed in colorectal cancer but rarely in the therapeutic range (2+ and 3+). There was no significant association with tumour grade, gender, localization of the primary tumour or survival. These data indicate that c-erbB-2 is unlikely to play a major role in the therapeutic management of colorectal cancer.

## Background

Colorectal cancer (CRC) is one of the most common malignancies in the western world [1]. The development of new cytotoxic agents (e.g. oxaliplatin and irinotecan) and surgical techniques have improved survival of patients with CRC. Once a patient becomes refractory to modern chemotherapeutic regimens no further treatment options are available. Recently the therapeutic armamentarium has been improved by the availability of monoclonal antibodies against the vascular endothelial – and epidermal growth factor receptor [2].

Her-2/neu oncogene is also a member of the tyrosine kinase family similar to the epidermal growth factor receptor (EGFR), HER-1, HER-3 and HER-4. HER-2 is located on chromosome 17q21 and encodes a 185 kD transmembran protein that lacks a natural ligand. HER-2 activation initiates signal cascades including the MAPK and PI3K/AKT pathways that are essential for cell proliferation and differentiation [3]. While the tyrosine kinase family receptors are found on normal cells, there is evidence that they are overexpressed in many types of tumours [4-6]. Clinically, c-erbB-2 amplification and/or overexpression has been associated with poor prognosis in a number of tumour types such as breast and ovarian cancer [4,7]. Pathologic specimens from the National Surgical Adjuvant Breast and Bowel Project protocol B-06 were reviewed and correlated with patients' outcome. Overall survival was decreased in all HER-2/neu-positive patients, and those patients having HER-2/neu overexpression with a good nuclear grade had a five-fold increase in mortality rate [8].

Overexpression of the HER-2/neu receptor is detected in 25–35% of human breast cancer patients [4,7]. Treatment of these patients with Herceptin^®^, an anti-HER-2 monoclonal antibody, has been shown to reduce tumour volume, to augment the effects of chemotherapy and to increase survival in primary and metastatic breast cancer [9,10]. The success of HER-2/neu directed therapy in breast cancer has lead to evaluations of protein expression and gene amplification in multiple tumour types, colorectal cancer among others. Herceptin^® ^has been shown to inhibit colony formation of the HCA-7 colon cancer cell line and HCA-7 tumour xenografts [11].

Conflicting data exist about the prevalence of HER-2/neu overexpression in colorectal cancer, which ranges from 0 to 83 % [12-16] as well as the relationship between HER-2/neu overexpression and clinicopathologic features like Dukes classification and survival.

The aim of our study was to determine the incidence of HER-2/neu positivity in colorectal cancers. Furthermore, we investigated the relationship of the HER-2/neu expression and patients' survival.

## Methods

### Patient information

This retrospective study involved 77 specimens of malignant colon lesions of patients who underwent elective surgery for colorectal cancer at the Medical University Vienna. All patients gave informed consent according to institutional guidelines (Medical University Vienna) prior to surgery. Tumours were collected in the Department of Pathology and staged according to TNM system and the Dukes classification [17,18]. Patients age, gender, site of primary tumour, degree of differentiation and stage are shown in Table 1. Patients were followed up every 3 months including clinical examination, laboratory tests including CEA, computed tomography of the abdomen and chest x-ray during the first 2 years after surgery and thereafter in 6–12 months intervals. Coloscopy was performed at 1-year intervals after resection of the primary.

None of the patients had received prior chemotherapy.

### Immunohistochemistry

HER-2/neu immunohistochemistry was performed using the Hercep-Test Kit (Dako, Glostrup, Denmark) according to the manufacturer's recommendations. 2 μm tissue sections were deparaffinized, rehydrated and placed in DAKO Epitope Retireval Solution for 40 minutes at 90°C, followed by cooling for 20 minutes at room temperature and treatment with peroxidase-blocking reagent. Afterwards slides were rinsed and incubated with the primary antibodies against HER-2/neu for 30 minutes followed by rinsing the slides and incubation with the DAKO Visualization Reagent for 30 minutes. After washing, the slides were incubated in diaminobenzidine for 10 minutes, counterstained with hematoxylin, de-hydrated and cover slipped.

### Scoring system

Evaluation of the results was done according to the criteria as recommended by the manufacturer using the scores from 0 to 3+. Score 0 is defended as no staining at all or membrane staining in < 10% of tumour cells. Score 1+is defined as faint/barely perceptable membrane staining in > 10% of tumour cells. The cells are only sarined in part of the membrane. 2+is defined as weak to moderate staining of the entire membrane in > 10% of the tumour cells. And 3+ is defined as strong staining of the entire membrane in > 10%. Score of 0 indicates a negative tumour, while scores of 1+, 2+ and 3+ were regarded as positive expression of c-erbB-2.

### Statistics

Expression of Her-2/neu was assessed with respect to TNM and Dukes' stage, degree of differentiation, site of primary tumour and patient gender using the chi-squared test. The effects of c-erbB-2 on survival were tested using Kaplan-Meier survival plots and analyzed using the log-rank test. Significance levels were set at p < 0.05. All statistical analyses were carried out using SPSS for Windows version 11.5.

## Results

A total of 77 patients with colorectal cancer were studied. For detailed patients' characteristics see Table 1. The median age of our patients, of whom 48 were male, was 52,5 years (range 30–83). The primary tumour site was the colon in 45 patients, the rectum in 30 patients and 2 patients had synchronous colonic and rectal cancer. Out of our 77 patients, 3 (4%) had Dukes A carcinoma, 8 (10%) Dukes B, 14 patients (18%) Dukes C and the majority (52 patients, 68%) Dukes D carcinoma. Histological grade included well (n = 3), moderate (n = 21) and poorly differentiated (n = 53) adenocarcinoma.

### Immunohistochemistry

The 77 colorectal samples were examined for the presence of Her-2/neu oncoprotein determined by immunhistochemistry. Out of this 77, 56 samples were Her-2/neu negative (70%). 20 (26%) showed a barely immunostaining, only 1 (1%) was moderately and 2 (3%) were strongly positive (Table 2).

### Her-2/neu expression and clinicopathological parameters

The expression of the c-erbB-2 protein was evaluated with respect to the patient's clinicopathological data. Her-2/neu staining (moderately and strongly positive) was only detected in primary tumours of patients with confirmed metastases in regional lymph nodes or distant organs (Dukes C or D) whereas tumours that were staged as Dukes A and B showed only no or barely p185^Her-2/neu ^immunostaining. There was a trend towards a decreasing frequency of positive c-erbB-2 tumours from colon to rectum but this was not significant (p = 0.251).

No relationship was found between membranous HER-2 expression and patients' gender, age or differentiation.

We also determined the association between the presence of Her-2/neu oncoprotein and survival. A difference was observed between positivity and survival although it was not significant. The median survival time of patients with positive p185^HER-2/neu ^immunostaining was 21 months versus 39 months in patients without HER-2/neu expression (p = 0.088).

## Discussion

The ErbB signaling network is known to influence a wide range of cellular processes, including proliferation, motility, and survival [19]. It is known that overexpression of EGFR often portends a worse prognosis [20,21]. The frequency of positivity appears to increase with clinical stage of disease. Her-2-neu positivity may thus represent a late event in the natural history of colorectal cancer and is associated with a worse prognosis. Overexpression of the HER-2/neu receptor is detected in 25–35% of human breast cancer [4,7] but the level and incidence of HER-2 overexpression in primary colon tumours appears to be different than those observed in breast cancer. Conflicting data exist about the prevalence of HER-2/neu overexpression in colorectal cancer which ranges from 0 to 83 % [12-16]. In our study, we examined 77 colorectal cancer tumour samples for the presence of Her-2/neu oncoprotein by immunhistochemistry. Out of these 77 samples, 56 (70%) were negative. Strong membranous HER-2 staining was only detected in 2 cases (3%). No correlation could be found between clinicopathological features and the HER-2/neu overexpression. Similar results are described in the study of Nathason et al [22]; they examined HER-2/neu gene amplification in 169 colon cancer specimens and HER-2/neu protein expression in 139 specimens, where they found HER-2/neu to be overexpressed in 5 cases (3.6%) and the gene to be amplified in 4 of these cases (2.4%). The HER-2/neu overexpression or the gene amplification was also not associated with any specific clinicopathological features [22]. In another study, strong membranous HER-2 staining was detected in only 5% of tumors in 96 primary human colorectal adenocarcinomas that also showed HER-2 gene amplification [23]. In contrast to these results are four studies who did report an association between Her-2/neu overexpression and advanced stage, decreased survival or both [14,15,24,25]. One of the studies by Kapitanovic et al [15] described a significant correlation between the epidermal abnormality degree and clinical parameters including Dukes' stage, relapse-free and postoperative survival.

Our study results indicating a very low rate of HER-2-/neu positivity and no correlation with clinicopathological features might be hampered by the small number of cases (77 specimens), However, the results are in agreement with same other larger patient series. In contrast to these data are the above mentioned four publications. The most likely reason for this divergency is the technical variability in the performance of immunhistochemistry. It is well known that there are pitfalls in immunostaining for HER-2/neu in breast cancer. Another reason may be due to the fact, that different antibodies have been used, stressing the importance of using standardized test systems most notably in case of therapeutic relevance of the results. Our results were all confirmed by a pathologist who is specialized in immunostaining for HER-2/neu and is a well known reference pathologist for this tumour entity. The inclusion of cytoplasmatic positivity in the Hercep-score in earlier papers may also be responsible for the conflicting results regarding the frequency of Her-2neu expression in colorectal cancer in the literature.

The c-erbB-e protein expression was observed in colorectal cancer but rarely in the therapeutic range (2+ and 3+). As known from studies in Her-2/neu metastatic breast cancer, Herceptin^®^, a HER-2 neutralizing antibody, is only effective in the therapeutic range. In a study by Ramanathan et al [26] Her-2/neu positive patients with advanced colorectal cancer should receive trastuzumab (Herceptin^® ^and irinotecan treatment. Of 138 screened patients Her-2/neu overexpression was only detected in 11 (8%; 2+ in 5 and 3+ in 6 patients), therefore the study was prematurely closed.

These data are similar to ours and indicate that c-erbB-2 is unlikely to play a major role in the therapeutic management of colorectal cancer. Therefore, further investigations of regimens involving trastuzumab seem not be useful.

## Conclusion

Overexpression of the HER-2/neu receptor is detected in 25–35% of human breast cancer [4,7] but conflicting data exist about the prevalence of HER-2/neu overexpression in colorectal cancer ranging from 0 to 83 %. In our study we analyzed 77 specimens of malignant colorectal cancer lesions. In only 4 % (3 specimens) the c-erbB protein expression was observed in the therapeutic range (2+ and 3+), 70% were Her-2/neu negative and 26% showed a barely immunostaining (1+). There was no significant association with tumour grade, gender, localization of the primary tumour or survival. These data indicate that c-erbB-2 is unlikely to play a major role in the therapeutic management of colorectal cancer.

## Competing interests

The author(s) declare that they have no competing interests.

## Authors' contributions

BS designed the study, performed the statistical analyses and wrote the manuscript. TG performed the statistical analyses of the data and interpretation of the data. FW scored the immunostained slides. WS and CZ participated in its design and helped to draft the manuscript. All authors read and approved the final manuscript.

## Pre-publication history

The pre-publication history for this paper can be accessed here:



## Figures and Tables

**Figure 1:**
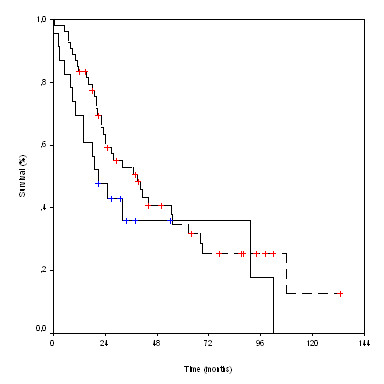
Survival according to Her-2/neu status. ---- HER-2/Neu neg.  HER-2/Neu pos.

**Table 1:** Patients characteristic

Gender	
Male	48
Female	29
	
Age (years)	
Median	52,5 years
Range	(range 30–83)
	
Dukes stage	
A	3 (4 %)
B	8 (10 %)
C	14 (18 %)
D	52 (68 %)
	
Tumour location	
Colon	45
Rectal	30
Both	2
	
Histological grade	
Well	3
Moderate	21
Poor	53

**Table 2:** Distribution of c-erbB-2 expression in colorectal cancer

Immunohistochemical score	n	%
0	54	70
1+	20	26
2+	1	1
3+	2	3

## References

[B1] Landis SH, Murray T, Bolden S, Wings PA (1999). Cancer statistics. CA Cancer J Clin.

[B2] Saltz LB, Meropol NJ, Loehrer PJ, Needle MN, Kopit J, Mayer RJ (2004). Phase II Trial of cetuximab in patients with refractory colorectal cancer that express the epidermal growth factor receptor. J Clin Oncol.

[B3] Schlessinger J (2000). Cell signaling by receptor tyrosine kinase. Cell.

[B4] Slamon DJ, Clark GM, Wong SG, Levin WJ, Ullrich A, McGuire WL (1987). Human breast cancer: correlation of relapse and survival with amplification of HER-2/neu oncogenes. Science.

[B5] Saranath D, Panchal RG, Nair R, Metha AR, Sanghavi VD, Deo MG (1992). Amplification and overexpression of epidermal growth factor receptor gene in human oropharyngeal cancer. Eur J Cancer.

[B6] Rusch V, Baselga J, Cordon-Carlo, Orazem J, Zaman M, Hoda S, McIntosh J, Kurie J, Dmitrovsky E (1993). Differential expression of the epidermal growth factor receptor and its ligand in primary non-small cell lung cancers and adjusent benign lung. Cancer Res.

[B7] Slamon DJ, Godolphin W, Jones LA, Holt JA, Wong SA, Keith DE, Levin WJ, Stuart SG, Udove J, Ullrich A, Press MF (1989). Studies of the HER-2/neu proto-oncogene in human breast and ovarian cancer. Science.

[B8] Paik S, Hazan R, Fisher ER, Sass RE, Fisher B, Realmond C, Schlessinger J, Lippman ME, King CR (1990). Pathologic findings from the National Surgical Adjuvant Breast and Bowel Project: prognostic significance of erbB-2 protein in primary breast cancer. J Clin Oncol.

[B9] Slamon DJ, Leyland-Jones B, Shak S, Fuchs H, Paton V, Bajamonde A, Fleming T, Eiermann W, Wolter J, Pegram M, Baselga J, Norton L (2001). Use of chemotherapy plus monoclonal antibody against HER2 for metastatic breast cancer that overexpress HER2. N Engl J Med.

[B10] Vogel CL, Cobleigh MA, Tripathy D, Gutheil JC, Harris LN, Fehrenbacher L, Salamon DJ, Murphy M, Novotny WF, Burchmore M, Shak S, Stewart SJ et, Press M (2000). Efficacy and safety of trastuzumab as a single agent in first-line treatment of HER2-overexpressing metastatic breast cancer. J Clin Oncol.

[B11] Mann M, Sheng H, Shao J, Williams CS, Pisacane PI, Sliwkowski MX, DuBois RN (2001). Targeting cyclooxygenase 2 and HER-2/neu pathways inhibits colorectal carcinoma growth. Gastroenterology.

[B12] Ross JS, McKenna BJ (2001). The Her-2/neu oncogene in tumors of the gastrointestinal tract. Cancer Invest.

[B13] Caruso ML, Valentini AM (1996). Immunhistochemical p53 overexpression correlated to c-erbB-2 and cathepsin D proteins in colorectal cancer. Anticancer Res.

[B14] Osako T, Miyahara M, Uchino, Inomata M, Kitano S, Kobayashi M (1998). Immunohistochemical study of c-erbB-2 protein in colorectal cancer and the correlation with patients survival. Oncology.

[B15] Kapitanovic S, Radosevic S, Kapitanovic M, Andelinovic S, Ferencic Z, Tavassoli M, Primorac D, Sonicki Z, Spaventi S, Pavelic K, Spaventi R (1997). The expression of p185(HER-2/neu) correlates with the stage of disease and survival in colorectal cancer. Gastroenterology.

[B16] McKay JA, Loane JF, Ross VG, Ameyaw MM, Murray GI, Cassidy J, McLeod HL (2002). C-erbB-2 is not a major factor in the development of colorectal cancer. Br J Cancer.

[B17] Beahrs OH (1992). Staging of cancer of the colon and rectum. Cancer.

[B18] Hermanek P, Altendorf A (1981). Pathol Res Pract.

[B19] Kirschbaum MH, Yarden Y (2000). The ErbB/Her family of receptor tyrosine kinase a potential target for chemoprevention of epithelial neoplasm. J Cell Biochem Suppl.

[B20] Baselga J, Mendelsohn J (1994). The epidermal growth factor receptor as a target for therapy in breast carcinoma. Breast Cancer Res Treat.

[B21] Baselga J, Mendelsohn J (1994). Receptor blockade with monoclonal antibodies as anti-cancer therapy. Pharmacol Ther.

[B22] Nathason DR, Culliford AT, Shia J, Chen B, D'Alessio MD, Zeng Z, Nash G, Gerald W, Barany F, Paty P (2003). HER-2/neu expression and gene amplification in colon cancer. Int J Cancer.

[B23] Half E, Broaddus R, Danenberg KD, Danenberg PV, Ayers GD, Sinicrope FA (2004). HER-2 receptor expression, localization, and activation in colorectal cancer cell lines and human tumors. Int J Cancer.

[B24] Lazaris AC, Theodoroporlos GE, Anastassopulos, Nakopoulou L, Panoussopoulus D, Papadimitriou K (1995). Prognostic significance of p53 and c-erbB-2 immunohistochemical evaluation in colorectal adenocarcinoma. Histol Histopathol.

[B25] Saeki T, Salomon DS, Johnson GR, Gullick WJ, Mandai K, Yamagami K, Moriwaki S, Tanada M, Takashima S, Tahara E (1995). Association of epidermal growth factor-related peptides and type I receptor tyrosine receptors with prognosis of human colorectal carcinomas. Jpn J Clin Oncol.

[B26] Ramanathan RK, Hwang JJ, Zamboni WC, Sinicrope FA, Safran H, Wong MK, Earle M, Brufsky A, Evans T, Troetschel M, Ealko C, Day R, Chen HX, Finkelstein S (2004). Low expression of HER-2/neu in advanced colorectal cancer limits the usefulness of trastuzumab (Herceptin) and irinotecan as therapy. A phase II trail. Cancer Invest.

